# Iohexol plasma clearance for measuring glomerular filtration rate: effect of different ways to calculate the area under the curve

**DOI:** 10.1186/s12882-021-02376-0

**Published:** 2021-05-05

**Authors:** Hans Pottel, Elke Schaeffner, Natalie Ebert, Markus van der Giet, Pierre Delanaye

**Affiliations:** 1 Department of Public Health and Primary Care, KU Leuven Campus Kulak Kortrijk, Etienne Sabbelaan 53, 8500 Kortrijk, Belgium; 2 Institute of Public Health, Charité Universitätsmedizin Berlin, Berlin, Germany; 3 Department of Nephrology and Intensive Care Medicine, Charité Universitätsmedizin Berlin, Berlin, Germany; 4 Department of Nephrology-Dialysis-Transplantation, University of Liège (ULg CHU), CHU Sart Tilman, Liège, Belgium; 5 Department of Nephrology-Dialysis-Apheresis, Hopital Universitaire Caremeau, Nimes, France

**Keywords:** Iohexol plasma clearance, Multiple samples, One sample, Measured GFR

## Abstract

**Background:**

Measuring glomerular filtration rate (GFR) using iohexol plasma clearance has been proposed as the preferred way for GFR determination. The extended multiple-sample protocol is based on fitting the full concentration-time decay-curve, and from the obtained fit-parameters, the area under the curve (AUC) and GFR (the injected dose divided by the AUC) were calculated. The goal of the current study is to evaluate the impact of different fitting procedures on the precision of GFR-results obtained from the full concentration-time curve, and compare these results with those obtained with simplified multiple-samples and single-sample protocols.

**Methods:**

The concentration-time curves of 8 samples at times 30, 60, 90, 120, 150, 180, 240 and 300 min after bolus injection of iohexol of 570 adults, aged 70+, from the Berlin Initiative Study (BIS), were analysed. The fit-parameters for the two-compartment model (double-exponential decay curve), and from these, the AUC and GFR were obtained with 8 different fitting procedures.

**Results:**

The two-compartmental non-linear least squares fitting procedure showed the best accuracy (541 out of 570 reported GFR-results were within 5% of the majority of the 8 fitting methods). The two-compartmental slope-intercept fitting procedure was not always applicable and the non-compartmental fitting procedures did not always allow to calculate the GFR. All correction formulas for the simplified late multiple-samples methods showed acceptable accuracy and precision with a preference for Ng’s correction formula (Lin’s CCC = 0.992, bias = 0.5 ± 2.5). Jacobsson’s iterative method was the best one-sample method, with Lin’s CCC = 0.983 and bias = − 0.6 ± 3.4.

**Conclusion:**

The fitting procedure has an important impact on the precision of the calculated AUC and GFR. The simplified late-sample protocols and one-sample methods did not suffer from fitting problems and showed acceptable equivalence when compared to the full compartment GFR-results.

**Trial registration:**

The “Berlin Initiative Study” is officially registered with the German Register for Clinical Studies (“Deutschen Register Klinischer Studien”(DRKS)) under registration number DRKS00017058, since April 12, 2019, and it is also visible on the WHO clinical trials registry platform (within the next 4 weeks after the registration date).

**Supplementary Information:**

The online version contains supplementary material available at 10.1186/s12882-021-02376-0.

## Background

Recently, it was suggested that iohexol, a non-ionic contrast agent, is most suited to replace inulin as the marker of choice for the determination of glomerular filtration rate (GFR) [1]. Plasma clearance measurement was chosen as the preferred procedure as it combines a reliable GFR determination with feasibility in the clinical setting. However, there are several protocols for the plasma clearance method using single and multiple samples. Also, the choice of the time-points may vary from protocol to protocol. A standardized protocol for the measurement of GFR is an important prerequisite for an unbiased and universally applicable reference standard procedure in the assessment of GFR [1, 2].

In the plasma clearance method, a bolus of an exogenous filtration marker is administered to a patient with subsequent blood sampling at specific time-points to measure the corresponding concentration. This procedure results in a concentration-time decay curve declining to zero once the exogenous marker is completely eliminated by the kidneys. The concentration-time curve is commonly expressed as a double exponential decay curve: c(t) = A_1_ exp.(−B_1_ x time) + A_2_ exp.(−B_2_ x time). Alternatively, the use of a three-compartment model has been proposed, but this requires a measurement protocol with extensive sampling and additional early time-points [3]. The fast component expresses the combination of the distribution or mixing of the exogenous marker in the body fluids and renal clearance, while the slow component represents the excretion phase and is solely related to renal clearance. It is assumed that the distribution phase completes within the first 120 min after bolus injection [4]. The c(t)-equation is fitted mathematically to obtain the coefficients A_1_, B_1_, A_2_ and B_2_. The area under the curve (AUC) is calculated from the obtained coefficients as AUC = A_1_/B_1_ + A_2_/B_2_. The GFR was then calculated as the injected dose divided by the AUC.

To our knowledge the effect of the fitting procedure to determine the coefficients of the double exponential decay and the calculated AUC and GFR has not been questioned yet. In most studies, the slow and fast compartment are considered separately, allowing the use of the slope-intercept method to obtain the fit parameters, but non-compartmental non-linear least squares fitting can also be applied to fit the c(t)-curve and to calculate the GFR. In other words, there are different mathematical procedures to calculate the AUC. The first aim of the study was to consider 8 different fitting procedures and investigate their effect on the reported calculated GFR, obtained from the same concentration-time decay curve.

The determination of the full plasma disappearance curve requires multiple blood samples (up to 10 samples), especially during the first hours [3, 5]. This procedure is cumbersome and prone to errors. Simplified procedures are preferred, which are based on the determination of the slow compartment only, including samples that are taken 120 min after injection, preferably limited to 3 or 4 time-points. As an additional step, the GFR calculated from the slow compartment model has to be corrected for the missing fast component. A number of correction formulas has been proposed to calculate the GFR from the slow compartment (GFR_S_) only [6–12]. The second aim of this study is to validate the accuracy of the correction formulas against GFR obtained from the complete plasma disappearance curve. Finally, we investigated the accuracy of two single-sample methods [13–15].

## Methods

The data from the Berlin Initiative Study (BIS) contains the disappearance of iohexol from blood in 570 study participants aged 70+ years. For each curve, 8 blood samples were drawn at fixed time-points 30, 60, 90, 120, 150, 180, 240 and 300 min after injection of 5 mL iohexol solution, containing 3235 mg of iohexol. The concentration was fitted to the double exponential decay curve. The measurement methods were previously described in detail [16].

### Fitting the double-exponential plasma clearance curve

We present 8 different fitting procedures, described in detail in the Additional file (section 4). These procedures are based on the slope-intercept (SI) method [5] or on non-linear least squares (NLLS) fitting using the Levenberg-Marquardt algorithm [17, 18]. Fitting can be compartmental (fitting slow and fast component separately, applicable to both SI and NLLS method) or non-compartmental (only applies for NLLS fitting). The SI-method log-transforms the mono-exponential decay (both for slow and fast component) to a linear equation obtaining its slope and intercept. A small twist on the SI-method is to use one common time-point for both the fast and slow component fit (120 min after injection was used here as the common time-point for the early and late regression models in the modified SI (mSI) method). The iterative NLLS fitting procedure can be unweighted (weights = 1) or may use relative weighting (weights = 1/Y^2^, where Y is the concentration), which puts less emphasis on the early time-points, rather than on the late time-points (higher concentrations get smaller weights). The NLLS-method can also be used when the double exponential decay is split up in the fast and slow component, analogous to the SI and mSI-method.

In summary, we used 8 different fitting procedures: 1) the SI-method (SI), 2) the modified SI-method (mSI), 3) the Split (S) NLLS no weights method (S-NLLS), 4) the Split NLLS 1/Y^2^ weights method (S-NLLS-w), 5) the modified Split NLLS no weights method (mS-NLLS), 6) the modified Split NLLS 1/Y^2^ weights method (mS-NLLS-w), 7) the NLLS no weights method (NLLS) and 8) the NLLS 1/Y^2^ weights method (NLLS-w). The first two procedures are based on the slope-intercept method. All other procedures are based on NLLS. The six first procedures use compartmental fitting, fitting fast and slow component separately. The last two procedures use non-compartmental fitting, not allowing to define the fast and slow component separately.

### Evaluating the fitting results

When fitting concentration-time decay curves, several problems can occur. First, the data at hand can lead to a “bad” fit, mostly due to errors in the measurement procedure (wrong time registration, errors in the measurement of iohexol in the blood samples, switching of samples, etc). *R*^2^ is not really appropriate to evaluate the goodness of fit in non-linear regression. However, in case of a monotone decreasing function, like the concentration-time curves in our study, it may still be useful to discriminate good from bad fits. We defined a ‘good’ fit when *R*^2^ > 0.975 (equivalent to *R* > 0.985, recommended in the BNMS guidelines [19] for the log-transformed slow mono-exponential decay), and a ‘bad’ fit when *R*^2^ < 0.900 (equivalent to *R* < 0.95). The fits with in-between *R*^2^-values were considered ‘acceptable’, but we visually inspected the residuals of the fitted results. “Bad fits” can sometimes be ‘solved’ by taking out a visually erroneous data-point, and the fitting procedure can be repeated without that data-point. Influence plots can help to identify the aberrant data-point.

Second, although the fit can be of acceptable quality, the calculations may not lead to a result. E.g. the SI-method was not always applicable due to negative residuals after subtracting the extrapolated slow component from the early concentrations. As the logarithm of negative residuals does not exist, the method is not applicable in these cases.

Third, the calculations may lead to an “unreliable” GFR-result. Cases with a reported GFR <  5 mL/min, were considered unreliable given that the subjects were from the general population, without known kidney problems. Although the fit is of acceptable quality, the concentration-time pattern may decay to a plateau value, leading to an AUC approaching infinity and thus a GFR approaching zero.

Fourth, the most problematic situation may occur when the fit is considered of acceptable quality and the calculated GFR seems reasonable, but, when applying different fitting methods, the results are very diverse. This variability in the reported GFR may raise questions about its validity.

### Simplified methods

Five different published correction equations to compensate for the absence of the “fast” first decay component were validated against the GFR obtained from the full concentration-time decay curve. The correction formulas can be linear (Chantler (C) = 0.87 x GFR_S_) [6], quadratic (Bröchner-Mortensen (BM) = 0.990778 x GFR_S_ – 0.001218 x GFR_S_^2^) [7, 20], or of the form: GFR = GFR_S_ / [1 + f x GFR_S_], with different values for f: *f* = 0.0012 from Ng (N) [8], 0.0017 from Fleming (F) [12] and 0.00185 x BSA^-0.3^ from BM-JØdal (BMJ) (BSA = Body Surface Area) [9]. In this evaluation, all GFR-values obtained from fitting the concentration-time curve are indexed for BSA, using GFR × 1.73/BSA [19]. We used the Dubois-Dubois BSA-formula for indexing GFR [21]. For the BM-model, we indexed by BSA before applying the correction [22]. Finally, the one-sample iterative Jacobsson procedure and the procedure proposed by Fleming were applied for the different late time-points and compared with the GFR-result obtained from the full concentration-time curve [13, 14].

### Statistics

The SI fitting procedure and mSI fitting procedure were programmed in SAS (SAS Institute Inc., Cary, NC, USA), using PROC REG for the linear regression and the non-linear least-squares (NLLS) procedures were executed using PROC NLIN. The NLLS-procedure used the “Marquardt” method and bounds were set for all fit-parameters to be positive. The relative difference (in %) within pre-specified percentage boundaries (5, 10, 15, 20%) between the eight fitting procedures was calculated to evaluate the concordance between the methods. To evaluate the performance of the correction formulas for the simplified slow-compartment methods and the one sample methods, Lin’s concordance correlation coefficient (CCC) was calculated as a measure of correlation and agreement with the GFR obtained using the S-NLLS procedure applied on the full disappearance curve as the reference procedure, except for the situations where GFR converged to zero. The root mean square error (rmse) is the root of the mean of squared differences calculated on the original scale and bias was defined as the average difference between the reference GFR obtained from the full disappearance curve and GFR obtained using the approximate methods. The standard deviation of the bias (SD) is a measure of precision for individual differences and the 95% Confidence Interval (CI) of the bias is a measure of precision of the mean difference.

## Results

### Comparison of models considering the full disappearance curve

The eight fitting methods were applied on the 570 decay curves. We identified 14 cases with unacceptable fit quality (*R*^2^ < 0.900). These curves were discarded from further analysis. We ranked the results of the 8 fitting procedures from smallest to largest per case, and calculated the mean of the 4 middle results to define a consensus result per case. We calculated the % deviation from the consensus for the results obtained from the different fitting methods (see Table 1).

**Table 1:** Relative difference (% deviation) between the GFR reported from a single full compartment method, compared to the consensus result for the 8 fitting methods. Only fits with *R*^2^ > 0.900 (*n* = 556) were considered

% deviation	SI	mSI	S-NLLS	mS-NLLS	S-NLLS-w	mS-NLLS-w	NLLS	NLLS-w
< 5%	531	502	541	541	517	538	425	384
5–10%	1	41	7	7	11	1	52	77
> 10%	24	13	8	8	28	17	79	95
NC	23	1	0	0	0	0	0	0
GFR < 5	0	0	5	5	9	8	31	25

*NC* not possible to calculate; GFR <  5 corresponds to AUC going to infinity (plateau value)

**Fig. 1:**
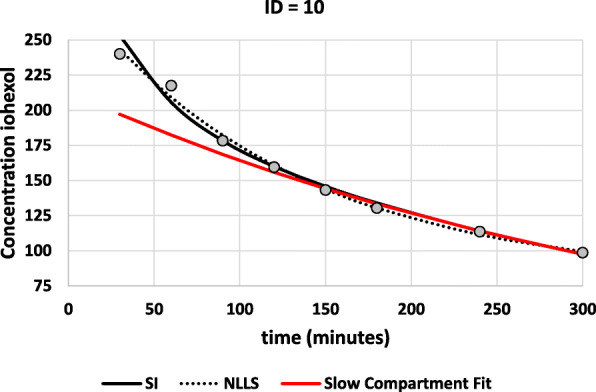
Result of the compartmental SI and non-compartmental unweighted NLLS fitting procedures. The slow compartment fit is also shown. The SI-method results in GFR = 37.5 mL/min/1.73m^2^, while the NLLS-method results in GFR = 0 mL/min/1.73m^2^ (because the curve ends in a plateau value)

The impact of the fitting procedure is further illustrated for patient with ID = 10 in detail in Fig. 1 (the time-concentration data and more details are available in the Additional file). The fitted curve using the SI-method and the unweighted NLLS-method resulted in GFR = 37.5 mL/min/1.73m^2^ and GFR = 0.0 mL/min/1.73m^2^ respectively. GFR (= Dose/AUC) equals zero because one of the B-coefficients = 0 for the NLLS-fitting method, and consequently the AUC becomes infinitely large. Another example is presented in the Additional file (section 1).

A method that could help to define the correct GFR is by adding random error to the data-points. We randomly generated 3000 new datasets based on the actual concentration-time decay data but introducing a maximum (random) error of ±2.5% for the time, concentration and injected dose. We then calculated the fit parameters and from this the AUC and GFR. We illustrated this method with two examples, applying the SI-method using the first 4 time-points for the early and the last 4 time-points for the late compartment (see the Additional file, section 2).

### Simplified multiple-sample protocols based on late compartment results

The reference GFR (calculated with the Split NLLS-method), which we here limited to the 541 cases having results within 5% of the consensus result, was plotted against the slow GFR (GFR_S_) (see the Additional file, section 3, Fig. S8), together with two of the correction models for the missing early compartment (Ng and Bröchner-Mortensen). The performance of the correction formulas was compared and the performance statistics are presented in Table 2. We calculated the f-values as (GFR_S_-GFR)/(GFR_S_ x GFR) and obtained a mean value of 0.0014 ± 0.0005. Based on the f-distribution (see the Additional file Fig. S9) and Table 2, the value of *f* = 0.0012, as proposed by Ng, seems to be the best choice for older adults. The C, BM, N, F and BMJ correction formulas show nearly equivalent performance (Table 2), demonstrating very high accuracy and precision for all correction methods, with bias between − 2.6 (C) and + 0.5 (Ng), RMSE varying from 2.50 mL/min/1.73m^2^ (Ng) to 3.61 mL/min/1.73m^2^ (C) and Lin’s CCC varying from 0.983 (C) to 0.992 (Ng).

**Table 2:** Performance statistics for the different correction formulas. The reference GFR was obtained with the S-NLLS-method on *n* = 541 subjects with deviations < 5% from the consensus

	C	BM	Ng	F	BMJ
LIN’s CCC	0.983	0.989	0.992	0.984	0.989
RMSE	3.61	2.85	2.50	3.37	2.76
Bias [95% CI]	−2.6 [−2.8;-2.4]	−0.9 [−1.1;-0.7]	0.5 [0.3;0.7]	−1.8 [− 2.0;-1.6]	−1.0 [− 1.2;-0.8]
SD	2.5	2.7	2.5	2.9	2.6

The slow GFR was obtained from the late decay curve with time-points 120, 150, 180, 240 and 300 min (GFR_S5_). The late decay curve was also calculated based on four time-points (leaving out 120 min (GFR_S4_)) and three time-points (leaving out 120 and 150 min (GFR_S3_)). The relationship between the calculated GFR from the full plasma disappearance curve (based on 541 measurements) and the GFR obtained from e.g. the BM-corrected slow GFR using 5, 4 and 3 time-points (180, 240 and 300 min) gave trend lines (with the intercept equal to zero) with slopes of 0.982 in all three cases, with *R*^2^-values of 0.983, 0.979 and 0.971, resp. In other words, the added value in terms of accuracy of using 5 (including 120 and 150 min) or 4 time-points (including 150 min) over the 3 time-points is very limited.

### Fleming’s and Jacobsson’s single-sample method

Finally, with the GFR-values of 541 subjects, the one-sample method proposed by Fleming and the method of Jacobsson, adapted by Eriksson was performed. The time-points of 120, 150, 180, 240 and 300 min were used to estimate the GFR from one sample. The performance statistics are presented in Tables 3 and 4. Jacobsson’s iterative method performed the best. The best overall time-point was 240 min, closely followed by 300 min. However, the performance depends on the GFR-level, with the best concordance with the GFR obtained from the full concentration-time curve, for the 300 min time-point when GFR < 60 mL/min/1.73m^2^, at the 240 min time-point when 60 < GFR < 90 mL/min/1.73m^2^ and at 180 min when GFR ≥ 90 mL/min/1.73m^2^ (Table 5).

**Table 3:** Performance statistics for the one-sample method of Jacobsson, adapted by Eriksson. The reference GFR was obtained with the S-NLLS-method on n = 541 subjects

Statistics	Time-point (minutes after injection)				
	120	150	180	240	300
LIN’s CCC	0.925	0.963	0.975	0.983	0.972
RMSE	7.60	5.28	4.25	3.46	4.40
Bias [95% CI]	−2.0 [−2.6;-1.4]	0.04 [−0.4;+ 0.5]	0.6 [0.2;0.9]	− 0.6 [− 0.9;-0.3]	− 2.3 [− 2.7;-2.0]
SD	7.3	5.3	4.2	3.4	3.7

**Table 4:** Performance statistics for the one-sample method of Fleming. The reference GFR was obtained with the S-NLLS-method on n = 541 subjects

Statistics	Time-point (minutes after injection)				
	120	150	180	240	300
LIN’s CCC	0.865	0.868	0.857	0.839	0.832
RMSE	9.18	8.90	9.20	9.78	9.98
Bias [95% CI]	−0.95 [−1.72;-0.18]	−2.12 [−2.85;-1.39]	− 2.97 [− 3.70;-2.23]	−4.05 [− 4.80;-3.30]	−4.21 [− 4.97;-3.44]
SD	9.1	8.7	8.7	8.9	9.1

**Table 5:** Number of concordant samples within 5%, 5–10 and >  10% according to S-NLLS GFR-category for the one-sample iterative Jacobsson method at 180, 240 and 300 min

	GFR-category (mL/min/1.73m^2^)			
180 min	0–30	30–60	60–90	> 90
< 5%	2	95	195	31
5–10%	2	54	70	15
> 10%	9	38	26	4
240 min	0–30	30–60	60–90	> 90
< 5%	1	135	233	26
5–10%	6	39	50	18
> 10%	6	13	8	6
300 min	0–30	30–60	60–90	90–120
< 5%	6	140	174	14
5–10%	6	43	107	25
> 10%	1	4	10	11
Total	13	187	291	50

## Discussion

The reference standard for measuring GFR is performed by loading and continuously infusing inulin and collecting timed urine samples and in-between blood samples. The method is complex, requires extensive technical assistance and a difficult chemical assay. Moreover, inulin is no longer available. As inulin disappears from the market, other filtration markers and protocols compete to replace it. Iohexol has been proposed as the most promising marker and plasma clearance measurement as the protocol of choice (1). The reference standard plasma clearance protocol determines the full concentration-time decay curve, a procedure that is not very convenient for the patient, as many blood samples are required. Simplified procedures have therefore been proposed, based on a few late samples (2 to 4) or only one late sample.

Only a few iohexol studies have been published including early (< 120 min) and late time-points [5, 16, 23, 24] and the ‘accuracy’ of the obtained GFR from the double-exponential decay curve has not been questioned so far. In the current study we have shown that the fitting procedure has significant influence on the obtained coefficients for the double exponential decay curve.

In case of the slope-intercept (SI) method, the AUC could sometimes not be calculated at all, while the NLLS procedure sometimes yielded zero values for one of the exponents (meaning that the curve ends on a plateau value), leading to an AUC of infinity, and consequently a GFR of zero. Although these curves, converging to a plateau, yielded the best fit statistics (smallest RMSE), such a plateau is physiologically impossible. However, when fitting procedures result in decay curves that end in a plateau, this may indicate that the last time-points are not ideally chosen, as the decay is going very slowly [25]. A possible solution would be to choose time-points (and their spacing) based on estimated GFR, but there are currently no recommendations or guidelines to help solving this issue. Actually, the goodness of fit was not our primary interest, but rather the reliability of the reported AUC and thus the GFR. To our knowledge there is no reliable and objective way to evaluate this, unless by increasing the confidence in the reported GFR when (nearly) all fitting methods report the same result. In the current study, only in 225 out of 570 cases (39.5%), the 8 methods gave GFR-values that were within 5% of each other, questioning the reliability of reported GFR-values when only one specific fitting procedure is selected, which is commonly done. The fitting procedure to obtain GFR is therefore a possible important source of error. We identified the split-compartment non-linear least squares method as the most robust method for reporting reliable GFR-values. In 541 out of 570 cases, this method was in concordance with the majority of the other methods. Thus, given the effect of the fitting procedure on the calculated GFR, the multiple-sample method may not be the preferred choice of direct GFR measurement, especially because simplified methods based on late multiple and single samples may show (nearly) equivalent accuracy and precision. Indeed, all eight fitting methods were able to accurately predict the concentrations for the late time-points (beyond 120 min), and thus the differences in reported GFR, obtained from the full concentration-time curves, were due to the variability in the predictions of the fast component (or the inability to accurately fit the fast component). Adding additional time-points (e.g. at 10 and 20 min, or even only one at 15 min) in the early (distribution) phase may (partially) solve this, but this requires additional discomfort for the patient. Moreover, studies with earlier time-points have reported the same fitting problems [24].

However, as the AUC of the slow compartment is contributing on average about 90% of the total area (see Additional file, section 3), correction formulas only have to correct for this last 10% which may explain why the different correction formulas do not differ much. The ‘worst’ correction formula for the data at hand is the Chantler correction formula, indicating that a linear correction is probably too simplistic. The ‘best’ correction formula for the older adult data at hand was the Ng correction formula, but with only a very small advantage over the other correction formulas. All correction formulas showed small bias and RMSE-values and can thus reliably be used. We also found no real difference between the slow GFR obtained from 5, 4 or 3 time-points beyond 120 min, illustrating that there is no value of adding time-points 120 and 150 min to the time-points 180, 240 and 300 min to determine the slow GFR. An important conclusion from this analysis is that the procedure limited to 180, 240 and 300 min combined with a correction formula is (nearly) as accurate as the reference standard procedure that fits the complete concentration-time decay curve. Moreover, the simplified procedure is more reliable in the cases where the results of the 8 different fitting procedures were very different.

As a further simplification of the plasma clearance method, the one-sample methods proposed by Jacobsson [13] and Fleming [15] were evaluated and showed surprisingly accurate results, especially when the time-point of 240 min (closely followed by the time-point of 300 min) was chosen. Although McMeekin showed that for patients of all ages the single sample technique developed by Fleming delivered the best accuracy and precision [26], we cannot confirm this finding, as in the current study, Jacobsson’s iterative method was the best performing one sample method. Choosing between 240 and 300 min, depending on the GFR-level (above or below 60 mL/min/1.73m^2^), even increases the one-sample accuracy. This confirms earlier findings comparing Jacobsson’s method with the simplified multiple-sample methods [27–29]. Gaspari et al. [29] found a good concordance (with an error ranging between − 5 and + 5%) in about 75% of the patients, whereas for the remaining 25% the prediction error ranged from − 22 to 40%. In the current study, we found that Jacobsson’s iterative method showed less than 5% difference with the reference standard GFR in 395/541 = 73% and less than 10% in 508/541 = 94% of the cases. This one-sample method can be extremely useful when large studies have to be conducted. Combined strategies to determine GFR, using the one-sample iohexol measurement together with demographic (age and body weight) and biomarker (serum creatinine and cystatin C) information can further improve the accuracy [30].

Limitation to the present study is the lack of independent reference standard renal clearance method, but previous studies comparing iohexol clearance with renal inulin clearance have shown a very high concordance between both methods [31]. A second limitation is the lack of persons with very low (< 30 mL/min/1.73m^2^) and very high GFR (> 90 mL/min/1.73m^2^). A third limitation is that we assumed no variability in the injected dose, registered time-values, and measured iohexol concentrations. All these possible sources of error will certainly have an influence on the fit-parameters, and thus on the calculated AUC and GFR. Simulation studies taking into account realistic variability in these variables may provide information on the expected variation in AUC and GFR, and the mean GFR-value of such simulation studies may provide the best estimate for the true mean GFR. McMeekin et al. found that all QC methods have poor sensitivity and a low positive predictive value for clinically significant errors in three point and single point sample methods, compared to the gold standard full compartment model GFR [32]. They concluded that QC methods in three point and single point sample methods cannot be relied on to ensure a robust measurement of GFR. QC methods for the gold standard method are also limited to evaluating the goodness of fit. In the present study we demonstrate that simulation studies introducing random error in the injected dose, measured concentrations and time-points, may provide QC information in terms of the shape and width of the GFR-distribution (see Additional file, section 2).

In conclusion, we found that the multiple sample two compartment model is the preferred (reference) method, nonetheless quality control is necessary to avoid the possibility of errors and ensure accurate measurement. This study also provides supportive data showing that iohexol can be used reliably in a simplified plasma clearance protocol, using only late time-points (e.g. 180, 240 and 300 min, with the possibility to add another late sample if a low GFR is expected), based on formulas to correct for the absence of the fast component, to directly measure GFR. Moreover, a slightly less accurate method using only one late sample is a valid alternative. The major limitation of these simplified methods is the lack of QC methods to evaluate the accuracy of the calculated mGFR.

## Supplementary Information

**Additional file 1: Table S1**. Time and concentration for ID = 10. **Fig. S1**. Result of the compartmental SI and non-compartmental unweighted NLLS fitting procedures. The slow compartment fit is also shown. **Fig. S2**. Squared residuals on the original scale for the NLLS and SI fitting methods (for ID = 10). **Table S2**. Fit statistics for example ID = 10 obtained with 8 different fitting procedures. **Table S3**. Fit parameters for example ID = 10 for the different fitting methods. **Table S4.** Time and concentration values for ID = 17. **Fig. S3**. Result of the compartmental SI and non-compartmental unweighted NLLS fitting procedures. The slow compartment fit is also shown (red curve). **Fig. S4**. Squared residuals for example ID = 17. **Table S4**. Fit statistics for example ID = 17 obtained with 8 different fitting procedures. **Table S5**. Fit parameters for example ID = 17 for the different fitting methods. **Table S6**. Reported GFR-values for two specific cases (NE = Not Estimable). **Fig. S5**. The concentration-time curves for the cases ID = 45 and 379. The red line is the fitted curve using the NLLS-method. **Fig. S6**. Distribution of GFR-results for ID = 45 obtained from the SI-method, based on 3000 new datasets derived from the original dataset by adding random error. **Fig. S7**. Distribution of GFR-results for ID = 379 obtained from the SI-method, based on 3000 new datasets derived from the original dataset by adding random error. **Fig. S8**. GFR against Slow GFR (GFR_S_) with some correction formulas: Ng (solid black curved line) and Bröchner-Mortensen (dotted line). Diagonal = identity line. **Fig. S9**. Distribution of f-values calculated for 541 GFR-values from the current dataset. The vertical lines correspond to the f-values proposed by Ng (*f* = 0.0012) and Fleming (*f* = 0.0017). The mean value was 0.0014 (SD = 0.005) and median was 0.0013. The mean value of *f* = 0.00185 x BSA^-0.3^ (Bröchner-Mortensen-Jødal) was 0.00154 with a range of [0.00142–0.00167].

## Data Availability

The data can be made available from Elke Schaeffner or Natalie Ebert on reasonable request. Study protocol and data set available from ES (e-mail, elke.schaeffner@charite.de). Statistical code available from HP (e-mail, hans.pottel@kuleuven.be).

## Abbreviations

GFR: Glomerular filtration rate
AUC: Area under the Curve
GFR_S_: Glomerular filtration rate calculated from the slow compartment only
BIS: Berlin Initiative Study
SI: Slope Intercept (method)
mSI: Modified slope intercept (method)
NLLS: Non-linear least squares (method)
NLLS-w: Weighted non-linear least squares (method)
S-NLLS: Split non-linear least squares (method)
S-NLLS-w: Split weighted non-linear least squares (method)
mS-NLLS: Modified split non-linear least squares (method)
mS-NLLS-w: Modified split weighted non-linear least squares (method)
C: Chantler correction
BM: Bröchner-Mortensen correction
N: Ng correction
F: Fleming correction
BMJ: Jodal Bröchner-Mortensen correction
BSA: Body surface area
CCC: Concordance correlation coefficient
RMSE: Root mean square error
SD: Standard deviation
CI: Confidence interval
*R*^2^: Coefficient of determination
