# Supplementary material for: Iohexol plasma clearance for measuring glomerular filtration rate: effect of different ways to calculate the area under the curve
Source: BMC Nephrol. 2021 May 5;22:166. doi: 10.1186/s12882-021-02376-0 (PMC8101203; doi:10.1186/s12882-021-02376-0)
Supplement: Supplementary file 1 — Additional file 1: Table S1. Time and concentration for ID = 10. Fig. S1. Result of the compartmental SI and non-compartmental unweighted NLLS fitting procedures. The slow compartment fit is also shown. Fig. S2. Squared residuals on the original scale for the NLLS and SI fitting methods (for ID = 10). Table S2. Fit statistics for example ID = 10 obtained with 8 different fitting procedures. Table S3. Fit parameters for example ID = 10 for the different fitting methods. Table S4. Time and concentration values for ID = 17. Fig. S3. Result of the compartmental SI and non-compartmental unweighted NLLS fitting procedures. The slow compartment fit is also shown (red curve). Fig. S4. Squared residuals for example ID = 17. Table S4. Fit statistics for example ID = 17 obtained with 8 different fitting procedures. Table S5. Fit parameters for example ID = 17 for the different fitting methods. Table S6. Reported GFR-values for two specific cases (NE = Not Estimable). Fig. S5. The concentration-time curves for the cases ID = 45 and 379. The red line is the fitted curve using the NLLS-method. Fig. S6. Distribution of GFR-results for ID = 45 obtained from the SI-method, based on 3000 new datasets derived from the original dataset by adding random error. Fig. S7. Distribution of GFR-results for ID = 379 obtained from the SI-method, based on 3000 new datasets derived from the original dataset by adding random error. Fig. S8. GFR against Slow GFR (GFRS) with some correction formulas: Ng (solid black curved line) and Bröchner-Mortensen (dotted line). Diagonal = identity line. Fig. S9. Distribution of f-values calculated for 541 GFR-values from the current dataset. The vertical lines correspond to the f-values proposed by Ng (f = 0.0012) and Fleming (f = 0.0017). The mean value was 0.0014 (SD = 0.005) and median was 0.0013. The mean value of f = 0.00185 x BSA-0.3 (Bröchner-Mortensen-Jødal) was 0.00154 with a range of [0.00142–0.00167]. [file 12882_2021_2376_MOESM1_ESM.docx]

Additional File

Iohexol plasma clearance for measuring glomerular filtration rate: effect of different ways to calculate the area under the curve

Hans Pottel^1^, Elke Schaeffner^2^, Natalie Ebert^2^, Markus van der Giet^3^, Pierre Delanaye^4,5^

^1^Department of Public Health and Primary Care, KU Leuven Campus Kulak Kortrijk, Kortrijk, Belgium.

^2^ Institute of Public Health, Charité Universitätsmedizin Berlin, Berlin, Germany.

^3^Department of Nephrology and Intensive Care Medicine, Charité Universitätsmedizin Berlin, Berlin, Germany

^4^Department of Nephrology-Dialysis-Transplantation, University of Liège (ULg CHU), CHU Sart Tilman, Liège, Belgium.

^5^Department of Nephrology-Dialysis-Apheresis, Hopital Universitaire Caremeau, Nimes, France

**Contents**

[**1.** **Examples of the effect of the fitting procedure on the calculated AUC** 2](#_Toc69199497)

[**1.1.** **Example ID = 10** 2](#_Toc69199498)

[**1.2.** **Example ID = 17** 5](#_Toc69199499)

[**2.** **Adding random error to the data-points** 9](#_Toc69199500)

[**3.** **Correlation between slow and total area** 11](#_Toc69199501)

[**4.** **Fitting the plasma clearance curve** 14](#_Toc69199502)

[**4.1.** **The slope-intercept method (SI-method)** 14](#_Toc69199503)

[**4.2.** **The modified slope-intercept method (mSI-method)** 16](#_Toc69199504)

[**4.3.** **Unweighted and weighted non-linear least squares (NLLS)** 16](#_Toc69199505)

[**4.4.** **Overview of fitting procedures** 19](#_Toc69199506)

[**5.** **References** 19](#_Toc69199507)

# **Examples of the effect of the fitting procedure on the calculated AUC**

## **Example ID = 10**

The original time and concentration data for this example are shown in Table S1.

**Table S1. Time and concentration for ID = 10.**

| **Time (min)** | **concentration** |
| --- | --- |
| 30 | 239.9117 |
| 60 | 217.3945 |
| 90 | 178.2150 |
| 120 | 159.4682 |
| 150 | 143.1718 |
| 180 | 130.3613 |
| 240 | 113.6223 |
| 300 | 98.6295 |

When fitting these data with the NLLS (no weights) procedure, the following fit-parameters were obtained: A_1_ = 57.2113, B_1_ = - 0.00076, A_2_ = 227.4, B_2_ = 0.00699, , which resulted in a negative AUC = -42745.8, which is physically impossible. When setting bounds requiring all fit-parameters to be positive, the new fit-parameters were: A_1_ = 79.0425, B_1_ = 1E-8, A_2_ = 206.9, B_2_ = 0.00773. This resulted in an AUC = ∞, and thus GFR = 0. The influence plot showed that observation #2 had the largest influence on the fitting parameters for the NLLS (unweighted) method.

When the observed and predicted concentrations are plotted on the original scale, the results of the fitting procedures can be inspected visually (see Figure S1). As an example, we show the fitting results for the patient with ID = 10, using the SI-method (solid line) and the unweighted NLLS-method (dotted line). The SI-method fits the slow compartment first to the late time-points (here 120, 150, 180, 240 and 300 min) (see red curve, which is extrapolated to the early time-points) and then fits the difference between the slow compartment extrapolation and the observed concentrations for the fast component, resulting in the combined equation shown by the solid line in Figure S1. The area in-between the red curve and the solid curve is the area of the fast component. The area under the red curve is the area for the slow component (which should be extrapolated to infinite time). This example also illustrates that the correction for the absence of the area from the fast component is small (usually smaller than 10%).

The contributions of each point to the SS_r_ are presented in Figure S2 for this example. Note that we here calculate the squared residual on the original scale, whereas the SI-method minimized the SS_r_ on the log-scale, while the NLLS-method minimized the SS_r_ on the original scale. What you can observe is that the NLLS-method better fits the fast part of the curve than the SI-method (smaller squared residuals). The major difference is in the fast part, not in the slow part.

The total SS_r_ for this example is 336.91 (see table S2) for the SI-method and 102.28 for the unweighted NLLS method. From figure S2, it can be seen that the two first time-points are contributing 314.25 (93.3%) to the SS_r_ for the SI-method, while this is only 77.85 (76.1%) for the first two points in the NLLS method. In both cases, the three early time-points contribute about 92-93% to the total SS_r_, demonstrating that the slow part is much better described by the model than the early part. In Table S2, the fit statistics are presented for the 8 different methods for example ID = 10.


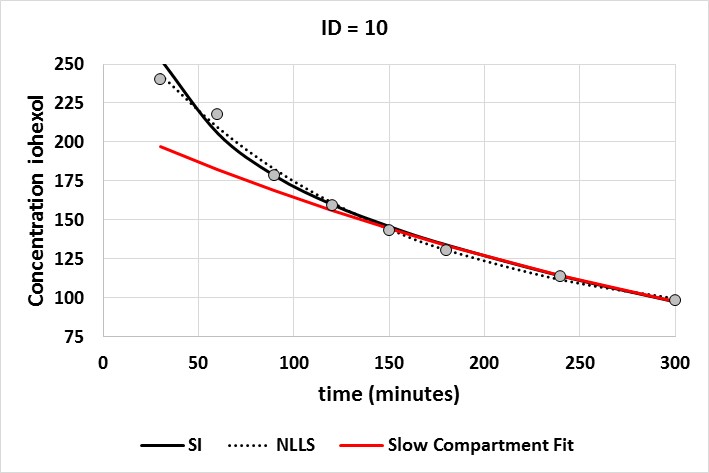


Figure S1. Result of the compartmental SI and non-compartmental unweighted NLLS fitting procedures. The slow compartment fit is also shown.


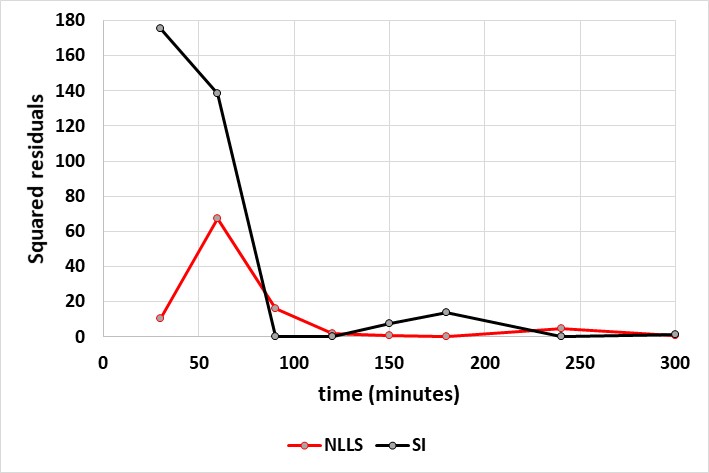


Figure S2. Squared residuals on the original scale for the NLLS and SI fitting methods (for ID = 10)

Table S2. Fit statistics for example ID = 10 obtained with 8 different fitting procedures

|  | SI | mSI | S-NLLS | mS-NLLS | S-NLLS-w | mS-NLLS-w | NLLS | NLLS-w |
| --- | --- | --- | --- | --- | --- | --- | --- | --- |
| SS_r_ | 336.91 | 1101.03 | 201.91 | 195.46 | 256.53 | 213.20 | 102.28 | 108.46 |
| R² | 0.9803 | 0.9356 | 0.9882 | 0.9886 | 0.9850 | 0.9875 | 0.9940 | 0.9937 |
|  |  |  |  |  |  |  |  |  |
| SS_E_ | 314.26 | 1084.08 | 120.59 | 127.72 | 234.17 | 181.29 | 94.15 | 104.15 |
| SS_L_ | 22.65 | 16.95 | 81.32 | 67.74 | 22.36 | 31.91 | 8.13 | 4.31 |
| Rank Best Fit | 7 | 8 | 4 | 3 | 6 | 5 | 1 | 2 |

SS_r_ = SS_E_ + SS_L_ (early and late SS) (the smaller the better)

The goodness of fit can be judged from the SS_r_ (the smaller the better) or from the R² (the closer to 1 the better) and the 8 methods are ranked according to this criterion. The fitted curve matches best the observed concentrations for the NLLS-method.

Table S3. Fit parameters for example ID = 10 for the different fitting methods

| Method | A_1_ | B_1_ | A_2_ | B_2_ | AUC | GFR (mL/min) |
| --- | --- | --- | --- | --- | --- | --- |
| SI | 134.66 | 0.02926 | 213.23 | 0.00261 | 86380 | 37.5 |
| mSI | 252.95 | 0.04187 | 213.23 | 0.00261 | 87819 | 36.8 |
| S-NLLS | 79.13 | 0.01981 | 215.84 | 0.00267 | 84777 | 38.2 |
| mS-NLLS | 82.12 | 0.02088 | 215.84 | 0.00267 | 84716 | 38.2 |
| S-NLLS-w | 114.34 | 0.02798 | 213.03 | 0.00260 | 85876 | 37.7 |
| mS-NLLS-w | 99.60 | 0.02478 | 213.03 | 0.00260 | 85809 | 37.7 |
| NLLS | 79.04 | 1.0E-08 | 206.94 | 0.00773 | 7904278430 | **0.0** |
| NLLS-w | 169.33 | 0.00970 | 120.80 | 0.00098 | 140544 | **23.0** |

However, the best fit resulted in an AUC ⇨∞, and consequently in a GFR ⇨ 0 (see Table S3). The second best fit, the weighted non-compartmental NLLS method (NLLS-w), resulted in a GFR = 23.0 mL/min. However, all other methods predicted a GFR closely around 37 – 38 mL/min. Note also that the contribution of SS_L_ to the total SS_R_ is (much) smaller than that of the SS_E_ for all fitting methods.

Note: Taking out the time-point “time = 60 min” resulted in a GFR = 31.7 mL/min and 32.1 mL/min for the NLLS and NLLS-w fitting methods. The SI-method (using 30, 90 and 120 min as early time-points) resulted in GFR = 36.7 mL/min.

## **Example ID = 17**

**Table S4. Time and concentration values for ID = 17**

| Time (min) | concentration |
| --- | --- |
| 30 | 264.5290 |
| 60 | 170.6695 |
| 90 | 143.0782 |
| 120 | 118.5563 |
| 150 | 102.9270 |
| 180 | 89.5715 |
| 240 | 67.937 |
| 300 | 51.058 |

When fitting these data with the NLLS (no weights) procedure, the following fit-parameters were obtained: A_1_ = 579.6, B_1_ = 0.0665, A_2_ = 214.5, B_2_ = 0.00483, which resulted in an AUC = 53138, and thus GFR = 60.9 mL/min. The influence plot showed that observation #3 had the largest influence on the fitting parameters for the NLLS (unweighted) method. Figure S3 shows the result of the fitting for the SI method and the NLLS method, and figure S4 illustrates the squared deviations between observed and predicted concentrations.


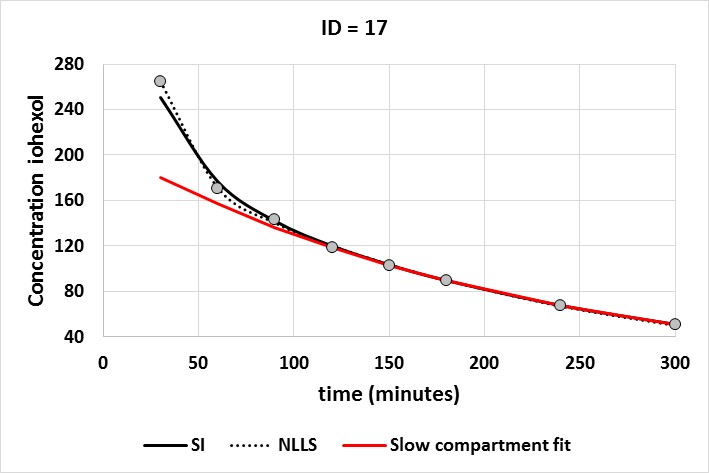


Figure S3. Result of the compartmental SI and non-compartmental unweighted NLLS fitting procedures. The slow compartment fit is also shown (red curve).


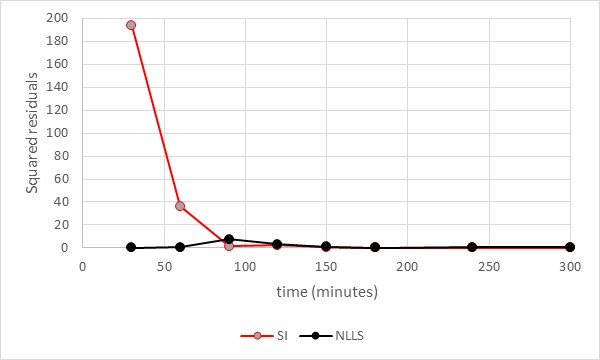


Figure S4. Squared residuals for example ID = 17

Table S4. Fit statistics for example ID = 17 obtained with 8 different fitting procedures

| Statistics | SI | mSI | S-NLLS | mS-NLLS | S-NLLS-w | mS-NLLS-w | NLLS | NLLS-w |
| --- | --- | --- | --- | --- | --- | --- | --- | --- |
| SS_r_ | 234.06 | 83.98 | 18.23 | 18.23 | 247.61 | 463.49 | 13.15 | 14.72 |
| R² | 0.9928 | 0.9974 | 0.9994 | 0.9994 | 0.9924 | 0.9857 | 0.9996 | 0.9995 |
|  |  |  |  |  |  |  |  |  |
| SS_E_ | 231.16 | 82.43 | 17.92 | 17.92 | 247.52 | 461.12 | 7.77 | 12.60 |
| SS_L_ | 2.90 | 1.55 | 0.32 | 0.32 | 0.08 | 2.36 | 5.38 | 2.12 |
| Rank Best Fit | 6 | 5 | 4 | 3 | 7 | 8 | 1 | 2 |

SSr = SS_E_ + SS_L_ (early and late SS)

Table S5. Fit parameters for example ID = 17 for the different fitting methods

| Method | A1 | B1 | A2 | B2 | AUC | GFR |
| --- | --- | --- | --- | --- | --- | --- |
| SI | 248.4 | 0.042151 | 207.6 | 0.00467 | 50359 | 64.2 |
| mSI | 312.2 | 0.046721 | 207.6 | 0.00467 | 51148 | 63.2 |
| S-NLLS | 461.9 | 0.05678 | 207.4 | 0.004663 | 52610 | 61.5 |
| mS-NLLS | 462.1 | 0.05679 | 207.4 | 0.004663 | 52611 | 61.5 |
| S-NLLS-w | 2562.6 | 0.11021 | 207.6 | 0.004669 | 67718 | **47.8** |
| mS-NLLS-w | 223.2 | 0.04215 | 207.6 | 0.004669 | 49762 | 65.0 |
| NLLS | 579.6 | 0.06647 | 214.5 | 0.004830 | 53138 | 60.9 |
| NLLS-w | 495.5 | 0.06013 | 210.5 | 0.004728 | 52772 | 61.3 |
| NLMIXED | 579.6 | 0.06647 | 214.5 | 0.004830 | 53133 | 60.9 |

In Table S4 the fit statistics for the example (ID = 17) are presented. From Table S4 it can be seen that the slow compartment fit parameters (A_2_ and B_2_) are approximately equal for the 6 split (compartmental) fitting procedures. The differences between the fitting results are mainly due to the fast compartment. The non-compartmental fitting procedure does not allow to define the slow and fast component separately, therefore the coefficients for A_2_ and B_2_ differ more from the split fitting procedures.

The fits can be ranked from 1 to 8 according to the smallest SS_r_ (or the greatest R²). The best fit, based on goodness of fit statistics, is given by the NLLS-method. Note that the mS-NLLS-w gives the worst fit statistics for this example. The reported GFRs are all relatively close to each other (61-65 mL/min), except for the S-NLLS-w method with 47.8 mL/min. Note also that the early compartment fit deviates much more from the measured data points (larger SS_E_) than the late compartment fit (much smaller SS_L_).

These two examples illustrate that depending on the methodology used to fit the 8 data-points, the GFR may vary considerably, and the method that best fitted the decay curve is not necessarily giving a reliable GFR-result (as in example ID = 10). The fact that the decay curve for ID = 10 obtained from the unweighted NLLS-procedure ends in a plateau-value may be a consequence of not having time-points later than 300 minutes in case of poor kidney function. It would be easy to jump to conclusions from this example, deciding that as the split methods give approximately the same results, we should discard the results from the non-compartmental fit procedures. The problem is that the situation can be very different, as is illustrated in another example (ID = 17) (Figure S3 and tables S4-S5). In this example, all but one method reports a GFR-value around 61-65 mL/min, except the S-NLLS-w method which reports a GFR of 47.8 mL/min. The NLLS-method is again the best fit method, and as the reported result is in line with 6 other methods, it is very probable that the GFR-value is 60.9 mL/min.

These two examples illustrate that the fitting procedure has an enormous impact on the calculation of the AUC, and consequently on the GFR. The fact that fitting procedures may report GFR-values that are very different also means that the uncertainty on the reported value may be large in case one fitting procedure has been selected. Researchers will most probably choose a specific fitting procedure (e.g. the SI-method) and will only switch to another fitting method in case the first method of choice did not result in a reasonable GFR-result. For example ID = 10, when the researcher has originally chosen the NLLS-method, it is easy to decide to switch to another fitting procedure, as the obtained result (GFR = 0) is unexpected and therefore probably considered unreliable. However, in case the researcher would have chosen the S-NLLS-w method as the preferred fitting method for our example ID = 17, then there would be no reason to switch method, and the reported GFR would probably be inaccurate.

It is important to note that this is always due to the fast component. The slow component could always be fitted (no exceptions) and the data were always acceptable from visual inspection. Also, from both examples it can be noted that the SS_r_ = SS_E_ + SS_L_ is mainly composed by the early component SS_E_ (which is calculated on 3 points only) and not by the SS_L_ (which is here calculated on 5 points). Thus, all methods are able to accurately fit the late component and have larger errors when fitting the early component.

This inaccurate fit of the early time-points can be solved by taking more early time-points, but this makes the method more complex and invasive.

# **Adding random error to the data-points**

We selected two cases (see Table S6) for which the SI-method was unable to fit the data. Although the fits were of acceptable quality, the reported GFR-results were very diverse for the different fitting methods.

Table S6. Reported GFR-values for two specific cases (NE = Not Estimable)

| ID | R² | SI | mSI | S-NLLS | mS-NLLS | S-NLLS-w | mS-NLLS-w | NLLS | NLLS-w |
| --- | --- | --- | --- | --- | --- | --- | --- | --- | --- |
| 45 | 0.971 | NE | 47.3 | 0.1 | 0.1 | 0.0 | 0.0 | 2.7 | 0.0 |
| 379 | 0.991 | NE | 56.7 | 21.8 | 21.8 | 0.0 | 0.0 | 54.5 | 54.4 |

R²-value is reported for the NLLS method


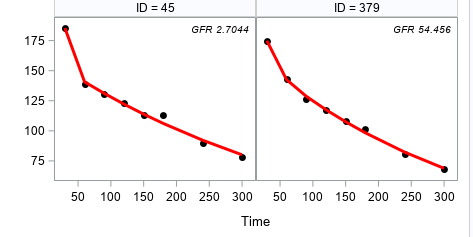


Figure S5. The concentration-time curves for the cases ID = 45 and 379. The red line is the fitted curve using the NLLS-method.

Using Microsoft Excel’s random number generator built-in function RAND () ( this function gives a random number between 0 and 1), we simulated 3000 new concentration-time decays per subject, with data around the original data, by randomly generating new time-points, new concentrations and a new injected dose, deviating from the original data by no more than ±2.5%, i.e. New Value = Original Value x (1 + r) with r = [-2.5 + 5.0 x RAND()]/100. We fitted each of these 3000 datasets using the Slope-Intercept method and calculated the GFR.

By adding error, the data is not completely the same and, for ID = 45, this resulted in 452 out of 3000 simulated datasets for which the SI-method provided a result. The mean GFR = 46.4 mL/min (median of 46.8 mL/min) was reported, which is quite close to the reported mSI-result (see table S6). The results ranged from 16.4 to 50.1. The distribution of the thus obtained GFR-results is shown in figure S6.


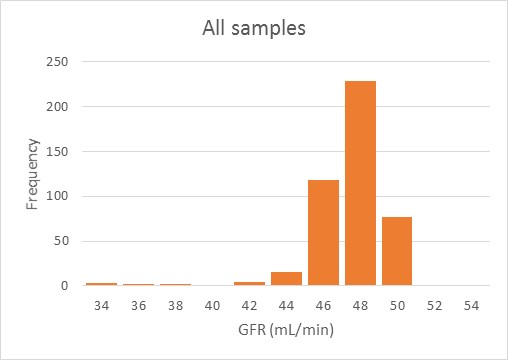


Figure S6. Distribution of GFR-results for ID = 45 obtained from the SI-method, based on 3000 new datasets derived from the original dataset by adding random error

As a second example we take ID = 379, for which the SI-method was also unable to calculate the GFR, the unweighted Split and modified Split method reported a GFR of 21.8 mL/min but the weighted Split and modified Split method reported a GFR of 0.0 mL/min; the mSI, NLLS and NLLS-w method reported a GFR of 56.7, 54.5 and 54.4 mL/min.

The simulations (based on the SI-method) resulted in a mean GFR = 55.1 mL/min (median of 55.7 mL/min) but with a range of possible results going from 6.0 to 60.5 mL/min. The distribution of 901 successful fits (out of 3000 simulated datasets) is shown in Figure S7.

Adding random error to the original data and generating the distribution of GFR-results from e.g. 3000 simulations, results in a mean or median GFR from the distribution which may be considered as a robust and reliable estimate of the true GFR, obtained from the full concentration-time decay curve.


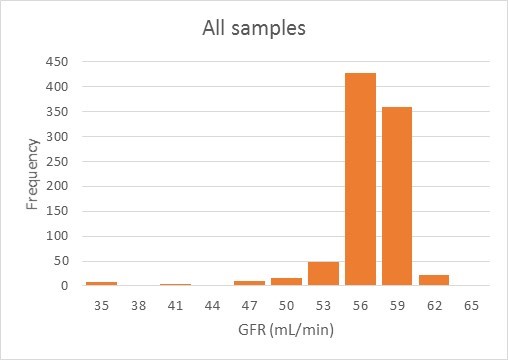


Figure S7. Distribution of GFR-results for ID = 379 obtained from the SI-method, based on 3000 new datasets derived from the original dataset by adding random error

# **Correlation between slow and total area**

There was a small but significant correlation between the slow Area and the fast Area (R = 0.313, p < 0.0001). However, the contribution of the fast area to the total area was, on average, equal to 9.7% (SD = 3.3%, range [1.2% - 17.0%]). This small contribution of the fast compartment to the total area, and thus to the calculated GFR, means that the correction is small and thus all correction formulas are very close to each other (less than 5 to 10%).

There exists a strong relationship between the slow Area and the total Area, calculated as Total Area = 1.0352 x Slow Area + 3347 (R² = 0.990). Forcing the intercept to zero results in a linear relationship: Total Area = 1.095 x Slow Area (R² = 0.986). From this simple linear relationship and the fact that GFR = Dose/AUC, it is easy to find that GFR = Dose/(1.095 x Slow Area) = 0.913 x GFR_S_, a linear correction formula close to the Chantler formula (GFR = 0.87 x GFR_S_).

Bröchner-Mortensen slightly corrected this linear relationship in a quadratic equation.

The reference GFR (calculated with the Split NLLS-method), which we here limited to the 541 cases having results within 5% of the consensus result, was plotted against the slow GFR (GFR_S_) (**Figure S8**), together with two of the correction models for the missing early compartment (Ng and Bröchner-Mortensen).
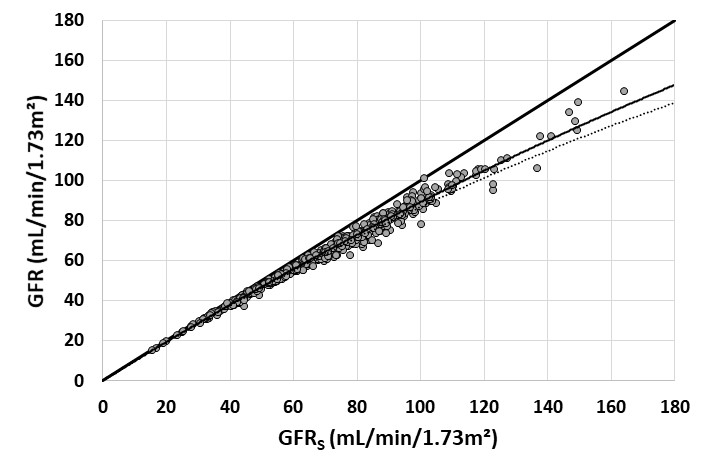


Figure S8: GFR against Slow GFR (GFR_S_) with some correction formulas: Ng (solid black curved line) and Bröchner-Mortensen (dotted line). Diagonal = identity line

The other correction formulas depend on the value of f which can be obtained as: f = [Slow GFR – GFR] / [Slow GFR x GFR] for each subject. The distribution of f-values is presented in Figure S9. We obtained a mean value of 0.0014 ± 0.0005. Based on the f-distribution (**Figure S9**) and Table 2, the value of f = 0.0012, as proposed by Ng, seems to be the best choice for older adults.

There was no important relation between f and age (R² = 0.011), f and BSA (R² = 0.106), f and BMI (R² = 0.028), and a small but significant difference of mean f-value between males and females (f = 0.00125 (males) vs f = 0.00151 (females), p < 0.0001).


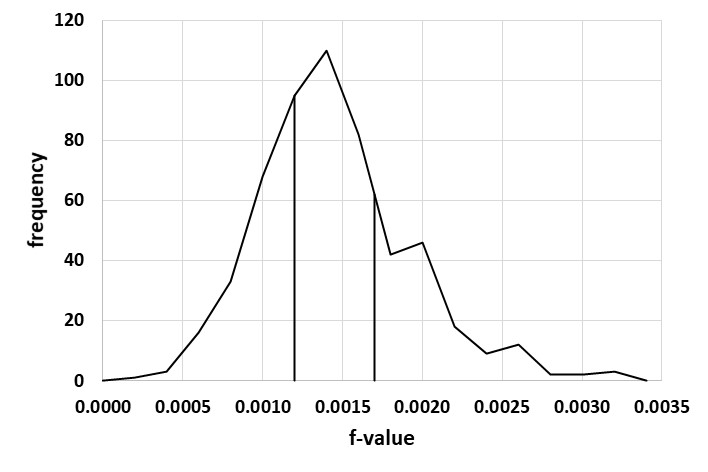


Figure S9. Distribution of f-values calculated for 541 GFR-values from the current dataset. The vertical lines correspond to the f-values proposed by Ng (f = 0.0012) and Fleming (f = 0.0017). The mean value was 0.0014 (SD = 0.005) and median was 0.0013. The mean value of f = 0.00185 x BSA^-0.3^ (Bröchner-Mortensen-Jødal) was 0.00154 with a range of [0.00142 – 0.00167].

# **Fitting the plasma clearance curve**

## **The slope-intercept method (SI-method)**

In the special case of a double exponential decay, which can be described as a two-compartmental model, the decay curve is considered as the sum of two mono-exponential decay curves, which are referred to as two compartments. Fitting can be done using the slope-intercept method (1) which is explained in three steps:

- Step 1. Fit the slow component on the late time-points (e.g. 120, 150, 180, 240 and 300 min), c(t) = A_2_ x exp(-B_2_ x time), by linear regression on the log-transformed c(t) against time, resulting in A_2_ and B_2_. To do this, the mono-exponential decay curve is log-transformed, resulting in ln[c(t)] = ln(A_2_) - B_2_ x time, which is a linear equation with slope (-B_2_) and intercept ln(A_2_). Slope and intercept are obtained by minimizing the sum of squared residuals SS_r_ = Σ [ln(c_i_) - ln(c_i_^*^)]², where c_i_ is the observed concentration and c_i_^*^ is the predicted concentration from the model. Note that this sum of squared residuals is calculated on the log-scale, which is not the same as the original scale.
  To evaluate the goodness of fit, the sum of squared residuals (SS_r_) and the explained variance R² = 1 – SS_r_/SS_T_, with SS_T_ the total sum of squares, (SS_T_ = sum of the squared differences between observed and mean of observed values, here on the log-scale) were calculated.
- Step 2. Calculate the subtractions or residuals (Res) for the early time-points (extrapolate the results from the late time-points to the early time-points): Res = c(t) – A_2_ x exp(-B_2_ x time) = A_1_ x exp(-B_1_ x time).
- Step 3. Fit the fast component by linear regression of the log-transformed residuals [ln(Res)] against time (for the early time-points only, e.g. 30, 60 and 90 minutes), resulting in A_1_ and B_1_. So, to obtain the fast part, one has to fit what has not been described by the slow part, which is the difference between the double exponential decay curve and the slow compartment model, here denoted by Res. So, by subtracting the predictions of the slow compartment model from the early time-points (earlier than 120 minutes), these data-points can be fitted with y = A_1_ exp(-B_1_ x time) (again, the fit is done on the linear equation which is obtained after taking the log-transform). The fitting parameters A_1_, B_1_, A_2_, B_2_ define the full concentration-time decay curve.

So, in brief, the method fits the slow compartment first (Step 1), and then subtracts the predicted early values from the initial fast compartment concentration values (Step 2), which can then be fitted separately (Step 3). In both cases (slow and fast component), the fit is based on a linear fit of the log-transformed concentrations.

The basic requirement for this method to work is that the subtractions of the extrapolated early concentrations from the initial concentrations are greater than zero (Step 2, the residuals ‘Res’). If this is not the case, the slope-intercept method cannot be used (as the logarithm of negative or zero values does not exist). In the specific case of the concentration-time decay curves of the 570 older subjects from the Berlin Initiative Study, this situation (negative subtractions) occurred in 33 cases (in 23 cases with acceptable R², in 10 cases the R² < 0.900), where the SI-method failed to perform the fitting.

The slope intercept method is thus based on two linear least-squares regressions on the log-transformed concentrations. This log-transformation changes the original scale to the logarithmic scale and consequently the contributions to the total sum of squares of observed minus predicted values. Actually, the slope-intercept method minimizes the sum of squares on the log-scale, not on the original scale. The spacing of the concentrations between the time-points therefore implicitly give weights (leverage) to each data-point. E.g. if the concentrations are equally spaced on the log-scale, all points get the same implicit weight (because they are equidistant), however, if the concentrations are equally spaced on the original scale, the implicit weights on the log-scale will not be the same (as the points are not equidistant on the log-scale, the leverage of far points are greater than for close points).

## **The modified slope-intercept method (mSI-method)**

A small twist on the SI-method is to use one common time-point for both the fast and slow component fit. In our specific situation, the mSI-method used time = 120 minutes as the common time-point for the early and late regression models. The common time-point mSI-method thus increases the number of time-points by one (here, from three to four) for the early component fit. Moreover, the residual concentration (or subtraction) for that time-point can artificially be set to zero (forced to zero, or to a very small value e.g. of 0.001 (as the log of zero does not exist)) which is argued by the fact that this is a common point with the slow compartment regression model. A further modification is that negative subtractions for the fast component are discarded, and the fit for the fast component is done with the remaining none-negative subtractions (if at least 2 points are remaining, including the zero residual of the common time-point).

## **Unweighted and weighted non-linear least squares (NLLS)**

An alternative fitting procedure would be to use non-linear curve fitting, based on the Levenberg- Marquardt algorithm. Least squares means that the sum of squared residuals (SS_r_) is minimized (= the least squares method). Residuals are the differences between observed and predicted values. So,

SS_r_ = Σ (y_i_ - y_i_^*^)²

where y_i_ = observed concentration and y_i_^*^ = predicted concentration from the equation.

In case all y_i_ = y_i_^*^ (the perfect model), the SS_r_ = 0. So, the aim of the fitting procedure is to minimize SS_r_. This is essentially the same as in the SI-method, except that the SS_r_ is calculated on the original scale, not on the log-scale.

When y_i_^*^ = A_1_ exp(-B_1_ x time) + A_2_ exp(-B_2_ x time) then the fitting procedure tries to find values for A_1_, B_1_, A_2_ and B_2_ to make SS_r_ as small as possible. As the equation is non-linear, the method is called Non Linear Least Squares fitting. Note that the method does not need to split up the double exponential decay curve into two compartments.

How does it work?

We start by plugging in some numeric starting values for A_1_, B_1_, A_2_ and B_2_. With these starting values, the SS_r_ is calculated. Of course, this SS_r_ will probably be quite large, so the algorithm tries to find new values for A_1_, B_1_, A_2_ and B_2_, so that the SS_r_ becomes smaller than the first SS_r_ calculated from the starting values. By the use of a gradient search algorithm the procedure iteratively recalculates new fitting parameters to decrease the sum of squared residuals. This iterative procedure stops when the final fit parameters are no longer changing and correspond to the smallest SS_r_ (least squares).

However, although non-linear least squares fitting leads to results for the fitting parameters, it may happen that the coefficients for the exponents are negative, which can be solved by setting bounds to the fit parameters (e.g. only allow positive fitting parameters).

The fitting procedure can be unweighted or may use weights for each data-point. A common weighting method, called relative weighting, is by using 1/Y² as weights, which puts less emphasis on the early time-points, rather than on the late time-points (higher concentrations get smaller weights). The SS_r_ is now defined as SS_r_ = Σ w_i_ x (y_i_ - y_i_^*^)² = Σ [(y_i_ - y_i_^*^)²/y_i_²], which is minimized. In the first procedure, there were no weights, or, you could say that the weights w_i_ = 1 for all data-points. In the second procedure, the weights are w_i_ = 1/y_i_², so the weights are not the same for all data-points, as the weight is smaller when y_i_² is larger. This weighted NLLS method will therefore give different final results as the non-weighted procedure.

It should be clear that each point will contribute to the SS_r_ when the observed concentration is not exactly equal to the predicted concentration. The differences are squared and summed to give the SS_r_. To illustrate the difference between weighted and unweighted NLLS, consider the following example. Suppose that the observed concentration at an early time-point is 500 and the model predicts a concentration of 450 for that time-point, then the contribution of that point to the SS_r_ will be (500-450)² = 50² = 2500. Suppose further that at a later time-point the observed concentration has dropped to 50, and you have the same % deviation (as for the high concentration), namely the predicted value is 45. So, the contribution to the SS_r_ for this data-point will be (50-45)² = 5² = 25. This is a factor 100 different from the contribution to the SS_r_ of the earlier time-point. So, one can imagine that the algorithm will try to match the high concentrations better than the low concentrations, because this will decrease the SS_r_ much more and the aim of the algorithm is to make the SS_r_ as small as possible. So, bringing the predicted concentration of the early time-point e.g. to 475 will reduce the contribution of that point to the SS_r_ from 2500 to (500-475)² = 25² = 625, a reduction of 2500 – 625 = 1875. Bringing the predicted concentration of the late time-point to e.g. 50 (which is the perfect fit for that time-point) will only reduce the contribution of that point to the SS_r_ from 25 to 0. Therefore, it should be clear that the unweighted fitting procedure will preferentially try to match the high concentrations, rather than the low concentrations. By weighing each data-point with 1/y_i_², this compensates the unequal contribution of each residual to the SS_r_. Now the contribution to the SS_r_ for the high concentration is (500-450)²/500² = 1/100 and for the low concentration it is (50-45)²/50² = 1/100, which is the same. This means that high and low concentrations are given the same weight in the SS_r_. There is no doubt that weighing will have an impact on the reported fitting parameters.

In case of a very slowly decaying concentration-time curve, this fitting procedure may result in a very small (close to zero) B-coefficient for the slow component, corresponding to reaching a plateau value, not allowing to calculate the GFR (actually the AUC would become infinite due to the plateau-value in the c(t)-curve). The corresponding GFR will tend to become zero, when the AUC ⇨∞.

The NLLS-method can of course also be used when the double exponential decay is split up in the fast and slow component. As in the scenario of the SI and mSI-method, the slow compartment can be fitted first, but now using NLLS-regression (either unweighted or weighted) followed by fitting the residuals of the fast component. Clearly, also the modified method (forcing the residual at time = 120 min to zero) can be applied.

For compartmental fitting, the SS_r_ was defined as the sum of the SS_E_ and SS_L_ (sum of squared residuals of the Early and Late data points). The best fit among the 8 fitting procedures is the one with the smallest SS_r_. To compare the fit results, the unweighted SS_r_ was calculated on the original scale for all fitting procedures. It is important to emphasize that the SI and mSI method minimize the SS_r_ on the log-scale for two compartments separately, and the weighted NLLS method minimizes the weighted SS_r_. Therefore, the comparison of the SS_r_ will always be in favour of the NLLS non-compartmental method, which directly minimizes SS_r_ on the original scale.

## **Overview of fitting procedures**

Summarized we defined 8 different fitting procedures:

| Name of method |  |  |
| --- | --- | --- |
| NLLS | Non-compartmental | Unweighted |
| NLLS-w | Non-compartmental | Weighted 1/Y² |
| Slope-Intercept (SI) | Two Compartmental | Split + Log-transform >> linear fit |
| mSI | Two Compartmental | Split + Log-transform + common point |
| S-NLLS | Two Compartmental | Split Unweighted NLLS |
| S-NLLS-w | Two Compartmental | Split Weighted NLLS |
| mS-NLLS | Two Compartmental | Split Unweighted NLLS + common point |
| mS-NLLS-w | Two Compartmental | Split Weighted NLLS + common point |

# **References**

1. Schwartz GJ, Furth S, Cole SR, Warady B, Muñoz A. Glomerular filtration rate via plasma iohexol disappearance: pilot study for chronic kidney disease in children. Kidney international. 2006; 69: 2070–2077
